# *Alhagi maurorum* Ethanolic Extract Rescues Hepato-Neurotoxicity and Neurobehavioral Alterations Induced by Lead in Rats via Abrogating Oxidative Stress and the Caspase-3-Dependent Apoptotic Pathway

**DOI:** 10.3390/antiox11101992

**Published:** 2022-10-07

**Authors:** Taghred M. Saber, Azza M. A. Abo-Elmaaty, Enas N. Said, Rasha R. Beheiry, Attia A. A. Moselhy, Fathy Elsayed Abdelgawad, Mariam H. Arisha, Taisir Saber, Ahmed Hamed Arisha, Esraa M. Fahmy

**Affiliations:** 1 Department of Forensic Medicine and Toxicology, Faculty of Veterinary Medicine, Zagazig University, Zagazig 44519, Egypt; 2 Department of Pharmacology, Faculty of Veterinary Medicine, Zagazig University, Zagazig 44519, Egypt; 3 Department of Veterinary Public Health, Faculty of Veterinary Medicine, Zagazig University, Zagazig 44519, Egypt; 4 Department of Histology and Cytology, Faculty of Veterinary Medicine, Zagazig University, Zagazig 44519, Egypt; 5 Department of Anatomy and Embryology, Faculty of Veterinary Medicine, Zagazig University, Zagazig 44519, Egypt; 6 Medical Biochemistry Department, Faculty of Medicine, Al-Azhar University, Cairo 11651, Egypt; 7 Chemistry Department, Faculty of Science, Islamic University of Madinah, P.O. Box 170, Madinah 42351, Saudi Arabia; 8 Department of Psychology, Faculty of Arts, Zagazig University, Zagazig 44519, Egypt; 9 Department of Clinical Laboratory Sciences, College of Applied Medical Sciences, Taif University, P.O. Box 11099, Taif 21944, Saudi Arabia; 10 Department of Animal Physiology and Biochemistry, Faculty of Veterinary Medicine, Badr University in Cairo (BUC), Cairo 11829, Egypt; 11 Department of Physiology, Faculty of Veterinary Medicine, Zagazig University, Zagazig 44519, Egypt

**Keywords:** lead, hepatotoxicity, oxidative stress, neurotoxicity, apoptosis, *Alhagi maurorum*

## Abstract

This work investigated the probable protective effect of an *Alhagi maurorum* ethanolic extract on the hepatotoxicity and neurotoxicity accompanied by neurobehavioral deficits caused by lead in rats. Rats in four groups were orally administered distilled water, ethanolic extract of *A. maurorum* (300 mg/kg BW daily), lead (100 mg/kg BW daily for 3 months), and lead + *A. maurorum* extract. The results demonstrated that lead exposure resulted in elevated locomotor activities and sensorimotor deficits associated with a decrease in brain dopamine levels. Moreover, lead exposure significantly increased liver function markers. In addition, the lead-treated rats exhibited extensive liver and brain histological changes and apoptosis. The lead treatment also triggered oxidative stress, as demonstrated by the increase in malondialdehyde (MDA) concentrations with a remarkable reduction in the activities of antioxidant enzymes, reduced glutathione (GSH) levels, and transcriptional mRNA levels of antioxidant genes in the liver and brain. Nevertheless, co-treatment with the *A. maurorum* extract significantly ameliorated the lead-induced toxic effects. These findings indicate that the *A. maurorum* extract has the ability to protect hepatic and brain tissues against lead exposure in rats through the attenuation of apoptosis and oxidative stress.

## 1. Introduction

Lead is a serious public health concern as it is one of the most ubiquitous poisonous heavy metals present in the environment. This is because of its environmental stability and extensive exposure. It can be found in various sources, including polluted drinking water, gasoline, batteries, paints, food cans, water pipes, conventional folk medications, cigarette smoke, and ceramic [1,2]. Lead could enter the body either via the ingestion of contaminated food or water or the inhalation of polluted dust and fumes [3]. Continuous exposure to lead, even at small doses, has been linked to hepatotoxicity [4], neurotoxicity [5], nephrotoxicity, testicular toxicity [6], and neurobehavioral alterations [7]. Lead toxicity stems mainly from the triggering of oxidative stress through the disruption of the pro-oxidant/antioxidant equilibrium and the upregulation of reactive oxygen species (ROS) [4].

*Alhagi maurorum* is considered as a hopeful medicinal plant because of the presence of phenolic compounds and flavonoids as its main components [8]. This plant belongs to the family Leguminosae and has several common names, such as camel thorn and Aqool [9]. It is used as a laxative, antirheumatic, anti-ulcerogenic, antipyretic, antiasthmatic, appetizer, expectorant, and diuretic agent in folk medicine [10,11]. *A. maurorum* possesses a wide variety of biological functions involving antiviral [12], anti-inflammatory [11], antioxidant [8], and anticarcinogenic [13] activities. Moreover, it has currently been reported to exert hepatoprotective and nephroprotective effects in animal studies [14,15,16]. To date, no studies have assessed the protective efficacy of *A. maurorum* against lead-induced hepatotoxicity and neurotoxicity. Hence, the goal of the current work was to assess the hepatoprotective and neuroprotective activity of an *A. maurorum* ethanolic extract against lead-mediated toxicity in rats through the estimation of behavioral changes, brain dopamine level, liver function, and oxidative stress markers, as well as the expression levels of apoptosis- and oxidative-stress-related genes in the liver and brain. Finally, histopathological and immunohistochemical investigations of the liver and brain were also carried out.

## 2. Materials and Methods

### 2.1. Chemicals and Tested Compounds

Lead acetate (C_2_H_3_O_2_)_2_, Pb, 99.6% purity) was obtained from El-Nasr Pharmaceutical Chemical Co., Cairo, Egypt. The leaves of *A. maurorum* were supplied by Sinai Moringa Co. for medicinal plants (El-Tur, South Sinai, Egypt). Other chemicals and reagents were purchased from Sigma–Aldrich Co. (St. Louis, MO, USA).

### 2.2. A. maurorum Ethanolic Extract Preparation

Preparation of the *A. maurorum* ethanol extract was performed according to the method of Rehman et al. [17]. This plant’s aerial parts were properly cleaned with distilled water before being dried in the shade. Approximately 500 g of dried plant powder was macerated in aqueous ethanol (70%) for 72 h and then filtered. The resulting ethanolic extract was subjected to concentration and evaporation under decreased pressure at 40 °C on a rotary evaporator (EYELA, CA-1111, Rikakikai Co., Tokyo, Japan). To assess the percentage yield, the concentrated extract was weighed and kept in a refrigerator (−4 °C) until needed.

### 2.3. Gas Chromatography/Mass Spectrometry Analysis (GC–MS) of A. maurorum Ethanolic Extract

The *A. maurorum* ethanol extract was subjected to GC–MS analysis (Agilent Technologies, Wilmington, DE, USA) equipped with a 7890B gas chromatograph and a 5977A mass spectrometer detector at Central Laboratories Network, National Research Centre, Cairo, Egypt. The different isolated constituents of the extract were identified by comparing their mass spectra with those stored in Wiley and NIST Mass Spectral Library data.

### 2.4. Experimental Animals

In this work, 40 mature male Sprague Dawley rats (10–12 weeks of age) weighing 200–230 g were supplied by the Laboratory Animals Research Center at the Faculty of Veterinary Medicine, Zagazig University, Zagazig, Egypt. The rats were housed under hygienic conditions in stainless steel cages under a photoperiod of 12 h light/dark with a controlled temperature (20–25 °C) and humidity of 60–65%. The animals were nourished on milk and barley with free access to tap water. Rats were also accustomed for 1 week before the beginning of the experiment. All experimental procedures were performed in accordance with the National Institutes of Health (NIH) guidelines for the Care and Use of Laboratory Animals in scientific investigations. The current protocol used in this investigation was approved by the Institutional Animal Care and Use Committee, Zagazig University, Zagazig, Egypt with an approval number: ZU-IACUC/2/F/174/2021. 

### 2.5. Experimental Design

The experimental animals were equally assigned into four groups, with each group encompassing ten rats. The control group was given distilled water orally. The Alhagi-treated group was gavaged with the ethanolic extract of *A. maurorum* dissolved in distilled water (300 mg/kg BW) daily for 3 months. The lead-treated group received lead acetate (100 mg/kg BW) dissolved in distilled water daily orally for 3 months. The lead + Alhagi-treated group was concomitantly treated with lead acetate (100 mg/kg BW) and the *A. maurorum* extract (300 mg/kg BW) as described above.

The dose of *A. maurorum* was selected based on an earlier report [8]. The dose for lead acetate was chosen based on a previous study [18]. 

### 2.6. Behavioral Response Assessment

To estimate the behavioral alterations caused by exposure to lead and/or *A. maurorum* ethanolic extract treatment, the rats were brought to their cages in the examination room and kept there for approximately 30 min before the test, to adapt them to the circumstances of the examination room. On the final day of the experiment, all tests were performed in the same examination room. The identity of the examined groups of animals was unknown to the tester. The various examined groups were subjected to behavioral assessments that included swimming performance, open field, grip strength, and inclined plane tests, as described below.

#### 2.6.1. Open Field Test

The rats were placed singly in the middle of the open field ring and behavioral responses were recorded for 5 min, starting 2 min after the rat was introduced in the test cage. The open field device was properly cleansed using ethyl alcohol (5%) prior to the placement of the next rat, to avoid the possibility of detection of the odor of previous rats. The behavioral parameters recorded in this experiment included rearing frequency (the number of times the animal stood on its hind legs), locomotion or ambulation frequency (the number of floor sections entered using two feet), grooming frequency (the number of grooming movements), and duration of freezing (immobility; i.e., elapsed time in seconds without any unexpected movements). In the open field, there was a central area or square that rats regarded as unsecured; therefore, the number of admissions into such area indicated the degree of anxiety-like behavior [19]. The observations were performed between 10:00 and 12:00 am.

#### 2.6.2. Swimming Performance Test

This test involved placing each rat in the middle of a glass tank for 5–10 s to monitor and score its swimming performance based on the position of its head and nose on the water surface, as described previously by Khalil et al. [7], as follows: 0, nose and head under the water surface; 1, nose under the water surface; 2, top of the head and nose at or over the water surface, with ears under the surface; 3, similar to score 2 but with the water line at the middle of the ear; and 4, similar to score 3 but with the water line at the base of the ears.

#### 2.6.3. Inclined Plane Test

The animals were placed horizontally on a flat surface (plane) with their heads toward the elevated side of the board [20]. A standard protractor was used to measure the inclined plane performance to the closest five grades. When the animal commenced sliding toward the back, the trial was terminated. Consequently, the trial was not time limited. The rat’s initial angle of sliding down was recorded. There were two trials performed on each rat, and the results were averaged. There was a 1 h interval between the two trials.

#### 2.6.4. Grip Strength Test

The strength of a rat’s forepaw was estimated by letting the rat grasp a wooden dowel (5 mm diameter) that was placed in a horizontal manner and lifted, allowing the animal to maintain its own weight, as explained by Andersen et al. [21]. The period required to let go of the grip was registered (in seconds). During this grip-strength testing, all animals attempted to hold the dowel. The results of each rat were averaged from two trials that were performed 1 h apart. 

### 2.7. Sample Collection

Blood samples were collected from the medial canthus of the control and the various experimental rats at the end of the dosage period and were left to clot for 30 min at room temperature. These samples were then centrifuged for 10 min at 3000 rpm to separate the serum. The resultant serum was preserved at −20 °C until they were used for the estimation of liver function and oxidative stress markers.

The rats from all experimental groups were weighed and euthanized by cervical dislocation. The brain and liver tissues were promptly removed and weighed after being rinsed with normal saline. The brain and liver specimens were rapidly frozen in liquid nitrogen and then kept at −80 °C for further RT-PCR analyses. Other liver and brain samples were kept at −20 °C for determination of oxidative stress parameters and the measurement of the dopamine neurotransmitter, whereas other specimens were fixed in 10% neutral buffered formalin for histopathological and immunohistochemical evaluations.

### 2.8. Biochemical Assays

#### 2.8.1. Evaluation of Liver Function Markers

The serum activities of alanine amino transferase (ALT) and aspartate aminotranserase (AST) were assayed using commercial kits supplied by Diamond Diagnostics (Cairo, Egypt).

#### 2.8.2. Assessment of Hepatic and Brain Oxidant/Antioxidant Status

Superoxide dismutase (SOD) and glutathione peroxidase (GPx) activities, and the levels of MDA (as a lipid peroxidation biomarker), and GSH were estimated in the liver and brain according to the methods described by Nishikimi [22], Gross et al. [23], Ohkawa et al. [24], and Beutler et al. [25], respectively. 

#### 2.8.3. Determination of Dopamine in the Brain

One gram of each brain specimen (cerebral cortex) was subjected to homogenization in 10 mL of potassium-phosphate-buffered saline (pH 7.4) using a tissue homogenizer. Subsequently, the samples were centrifuged for 10 min at 3000 rpm to obtain the supernatant, which was used for the estimation of the dopamine level using rat-enzyme-linked immunosorbent assay (ELISA) kits provided by CUSABIO, Wuhan, China (Cat. No. CSB-E08660r).

### 2.9. Analysis of Apoptosis- and Oxidative-Stress-Related Gene Expression

Extraction of total RNA from the liver and brain tissues was performed using the TRIzol (Invitrogen; Thermo Fisher Scientific, Fremont, CA, USA, Catalog No. 15596026) according to the manufacturer’s instructions. Total RNA (1 µg) was reverse-transcribed into cDNA using the HiSenScript™ RH (-) cDNA Synthesis Kit provided by iNtRON Biotechnology Co. (Seongnam, Korea), according to the manufacturer’s protocol, as documented previously by Arisha et al. [26] and Saber et al. [27]. For the analysis of gene expression, real-time RT-PCR was performed using aCFX96 real-time PCR detection system (CFX96; Bio-Rad, Hercules, CA, USA) with the TOPreal™ qPCR 2X PreMIX (SYBR Green with low Rox) supplied by Enzynomics Inc. (Daejeon, Korea) according to the instructions of the manufacturer. The PCR cycling conditions included a preliminary denaturation at 95 °C for 15 min, followed by 40 cycles of denaturation at 95 °C for 30 s and annealing at 60 °C for 60 s and a final elongation at 72 °C for 60 s. The oligonucleotide specific primers used here were manufactured by Sangon Biotech (Beijing, China) and are listed in Table 1. The relative fold changes in gene expression were calculated using the 2CT (cycle threshold) comparative method [28] after normalizing the expression levels of the studied target genes to that of the *GAPDH* gene.

### 2.10. Histopathological Investigations

The fixed brain and liver samples were dehydrated using increasing ethanol grades, cleared in xylene, and finally embedded in paraffin; subsequently, paraffin sections were obtained (4 μm thickness). These sections were stained with hematoxylin and eosin (H&E) as reported previously by Suvarna et al. [29]. The slides were examined using an Olympus BX61 light microscope (Tokyo, Japan).

For lesion scoring, five non-repeated, randomly selected microscopic fields (magnification, 40×) were examined in three different slides per animal/group. The mean values of the five microscopic fields examined were considered the final lesion score of each animal. The histopathological lesions observed in the liver, cerebellar, and cerebral cortices, and hippocampus in all examined groups were assessed using the following scoring method; 0, _1, _2, _3, _4, and _5 corresponding to no change, mild change, mild-to-moderate change, moderate change, moderate-to-severe change, and severe change, respectively [30].

### 2.11. Immunohistochemical Investigation of Bcl-2

Another set of liver and brain paraffin sections were used for the immunohistochemical detection of Bcl-2 using an anti-Bcl-2 monoclonal antibody (10C4) purchased from Thermo Fisher Scientific, Fremont, CA, USA (Catalog # 33-6100), together with the avidin–biotin–peroxidase method developed by Ramos-Vara et al. [31]. As an alternative to the primary antibodies used here, phosphate-buffered saline was utilized to prepare negative-control sections. For the quantitative assessment of Bcl-2 immunoexpression, five random and non-overlapping fields were selected (magnification, 40×) per section (30 images/group), and the immunoexpression was assessed by calculating the percentage of positive cells/image using the following score: negative-to-weak expression, <10%; mild expression, 10–25%; moderate expression, >25–50%; strong expression, >50–75%; and overexpression, >75% [32].

### 2.12. Statistical Analysis

All data were checked for normality using the Shapiro–Wilk test and for homogeneity of variance using Levene’s test. The current data were presented as the mean ± standard error (SE). The results were analyzed statistically using one-way analysis of variance (ANOVA). The mean values of the control and various experimental groups were compared using the post-hoc Duncan’s test. The IBM SPSS Statistics computer software (version 22) was used to perform the statistical analyses. A *p*-value < 0.05 was regarded as statistically significant.

## 3. Results

### 3.1. GC-MS Profile of A. maurorum Ethanolic Extract

**Figure 1:**
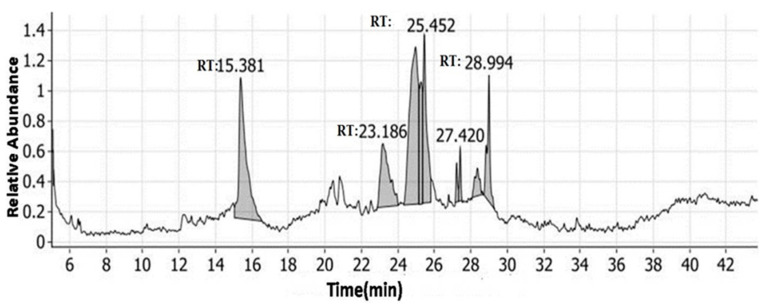
GC-MS chromatogram of *A. maurorum* ethanolic extract.

The GC-MS analysis of *A. maurorum* extract demonstrated the main ingredients, their relative percentage of the total peak area sum and retention times (Table 2 and Figure 1). The data indicated that the extract contained nine components, which included beta-D-glucopyranose, 1,6-anhydro-(levoglucosan) (28.91%), 4H-pyran-4-one, 2,3-dihydro-3,5-dihydroxy-6-methyl- (23.24%), 3-methyl-2-(2-oxopropyl)furan (13.56%), and 3-methyl-2-(2-methyl-2-butenyl)-furan (rosefuran) (12.84%).

### 3.2. Effect of the A. maurorum Extract on Body Weight Gain, and Relative Brain and Liver Weights in Lead-Exposed Rats

The lead-exposed group exhibited a significant decrease (*p* < 0.001) in body weight change compared with the control rats. In contrast, co-treatment with *A. maurorum* extract significantly mitigated (*p* < 0.001) this negative effect of lead by increasing the lowered body weight gain values in the lead + Alhagi-exposed rats compared with the lead-treated rats. However, no significant differences in relative liver and brain weights were observed among all of the studied groups (Table 3). 

### 3.3. Effect of the A. maurorum Extract on the Neurobehavioral Responses of Lead-Exposed Rats

**Figure 2:**
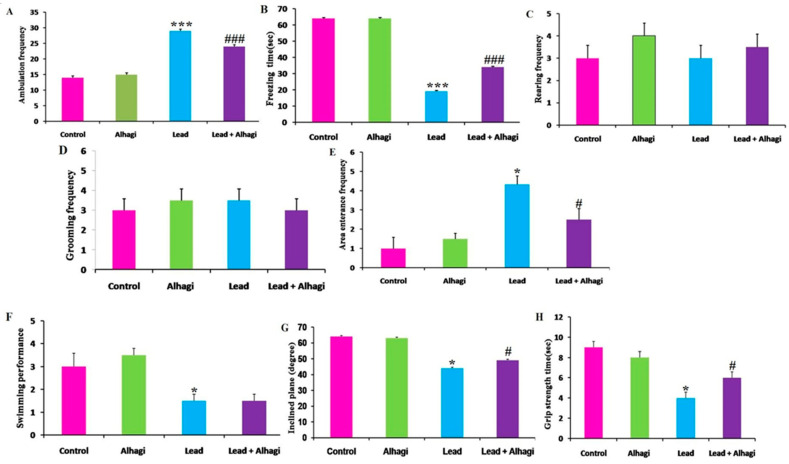
Effect of *A. maurorum* extract on open field test parameters (ambulation frequency (**A**), freezing time (**B**), rearing frequency (**C**), grooming frequency (**D**), and central area entrance frequency (**E**), swimming performance (**F**), inclined plane test (**G**), and grip strength time (**H**) in the lead-treated rats. Data were expressed as mean ± SE, *n* = 10 for each group. *, *** Significantly different compared to the control group at *p* < 0.05 and *p* < 0.001, respectively. #, ### Significantly different compared to the lead-exposed group at *p* < 0.05 and *p* < 0.001, respectively.

Lead exposure provoked a significant increment (*p* < 0.001) in the locomotor activity of the rats, as indicated by a marked elevation in ambulation frequency, which was linked to a reduction in the freezing time in the lead-intoxicated group compared with the control group. Conversely, simultaneous treatment with the *A. maurorum* extract significantly (*p* < 0.001) lowered the ambulation and increased the freezing time relative to the lead-intoxicated group, although it did not restore such parameters to the control values. There were no significant changes in grooming and rearing behaviors after the lead treatment. Furthermore, these rats entered the central area more frequently than did the control animals. In turn, this behavior was normalized by concomitant treatment with the *A. maurorum* extract in the lead + Alhagi-exposed group. In addition, significant (*p* < 0.05) decreases in swimming and inclined plane performance, as well as grip strength time, were detected in the lead-treated rats relative to the control group, which demonstrated that the lead-exposed rats had sensorimotor deficits. Conversely, simultaneous treatment with the *A. maurorum* extract resulted in a significant restoration of the inclined plane performance and grip strength time, but had no effect on swimming performance (Figure 2).

### 3.4. Effect of the A. maurorum Extract on Liver Function Markers in Lead-Treated Rats

The lead-treated group exhibited significantly increased (*p* < 0.001) serum AST and ALT activities relative to the control rats. In contrast, the lead + Alhagi- exposed rats showed a significant decrease (*p* < 0.001) in the activity of these enzyme biomarkers compared with the lead-treated rats. In turn, supplementation with the *A. maurorum* extract restored the AST activity to the normal level in the lead + Alhagi-treated group, but did not restore that of ALT to the control value (Table 4).

### 3.5. Effect of the A. maurorum Extract on Hepatic and Brain Oxidative Stress Biomarkers in Lead-Exposed Rats

Lead-exposed rats displayed a significant (*p* < 0.001) decrease in the activity of SOD and GPx, as well as in GSH concentration in the liver and brain, compared with the control rats. In contrast, concurrent treatment with the *A. maurorum* extract significantly (*p* < 0.001) enhanced the reduced values of such biomarkers, and restored them toward the normal values in the lead + Alhagi-exposed rats. In addition, a notable elevation (*p* < 0.001) in MDA concentration was observed in the liver and brain of lead-intoxicated rats compared with the control group. However, the lead + Alhagi-exposed group exhibited a significant decrease in the liver and brain MDA concentrations—although they were not restored to the control levels—compared with the lead-exposed rats (Table 4).

### 3.6. Effect of the A. maurorum Extract on the Brain Dopamine Level in Lead-Treated Rats

The concentration of dopamine was significantly (*p* = 0.001) reduced in the brain of lead-exposed rats compared with the control rats. Conversely, concomitant treatment with the *A. maurorum* extract in the lead-treated group significantly (*p* = 0.001) elevated the brain dopamine level, which was restored to the control values, in the lead + Alhagi-exposed group (Table 4).

### 3.7. Effect of the A. maurorum Extract on the Hepatic mRNA Expression of Apoptotic Marker and Antioxidant Genes in Lead-Exposed Rats

**Figure 3:**
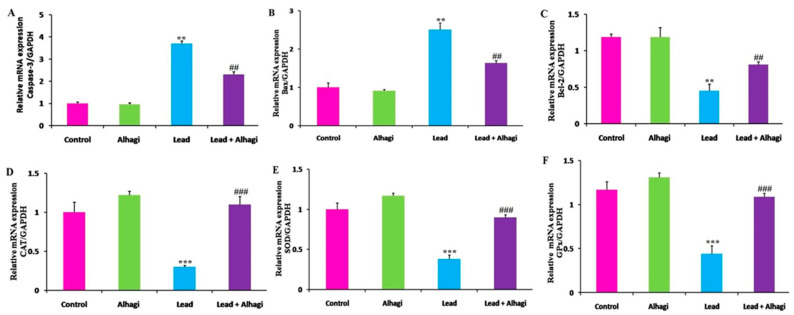
Effect of the *A. maurorum* extract (300 mg/kg BW, orally) on the hepatic mRNA expression of apoptosis-associated genes (**A**–**C**) and antioxidant genes (**D**–**F**) in rats treated with lead (100 mg/kg BW, orally) daily for 3 months. *Caspase-3* (**A**), *Bax* (**B**), *Bcl-2* (**C**), *CAT* (**D**), *SOD* (**E**), and *GPx* (**F**). Data were expressed as mean ± SE, *n* = 10 for each group. **, *** Significantly different compared to the control group at *p* < 0.01 and *p* < 0.001, respectively. ##, ### Significantly different compared to the lead-exposed group at *p* < 0.01 and *p* < 0.001, respectively.

Lead treatment led to significant (*p* < 0.01) upregulation of the hepatic mRNA expression of the pro-apoptotic *caspase-3 and Bax* genes, with marked downregulation of the anti-apoptotic *Bcl-2* gene, compared with the control rats. Conversely, oral supplementation with the *A. maurorum* extract significantly suppressed (*p* < 0.01) the hepatic expression of the *caspase-3 and Bax* genes and elevated the *Bcl-2* mRNA expression levels in the lead + Alhagi-exposed rats compared with the lead-intoxicated group. Although concomitant treatment with the *A. maurorum* extract considerably improved the hepatic apoptotic status, apoptotic markers were not restored to the control values in the lead + Alhagi-exposed group. Moreover, the transcriptional levels of antioxidant genes, including *CAT, SOD,* and *GPx,* were significantly downregulated (*p* < 0.001) in the liver of lead-intoxicated rats compared with the control group, whereas the transcriptional levels of these genes were markedly upregulated after *A. maurorum* extract co-administration, which restored them to the normal levels in the lead + Alhagi-exposed group (Figure 3).

### 3.8. Effect of the A. maurorum Extract on the mRNA Transcriptional Levels of Genes Related to Apoptosis and Oxidative Stress in the Brain of Lead-Exposed Rats

**Figure 4:**
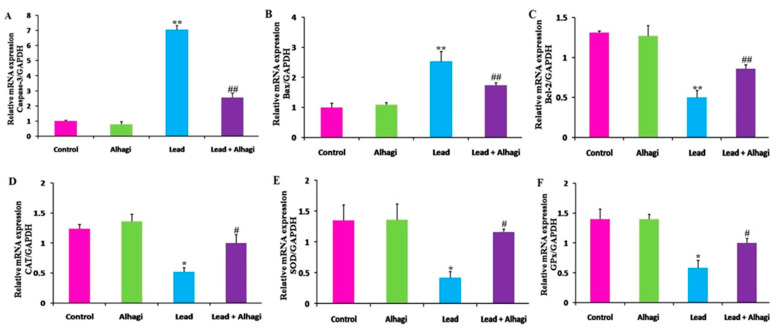
Effect of the *A. maurorum* extract (300 mg/kg BW, orally) on the mRNA levels of genes related to apoptosis (**A**–**C**), and oxidative stress (**D**–**F**) in the brain of rats treated with lead (100 mg/kg BW, orally) daily for 3 months. *Caspase-3* (**A**), *Bax* (**B**), *Bcl-2* (**C**), *CAT* (**D**), *SOD* (**E**), and *GPx* (**F**). Data were expressed as mean ± SE, *n* = 10 for each group. *, ** Significantly different compared to the control group at *p* < 0.05 and *p* < 0.01, respectively. #, ## Significantly different compared to the lead-exposed group at *p* < 0.05 and *p* < 0.01, respectively.

A significant (*p* < 0.01) elevation in the mRNA transcriptional levels of *caspase-3* and *Bax*, and a remarkable suppression of the *Bcl-2* mRNA expression levels, were noted in the brain of the lead-intoxicated group compared with the control group. In contrast, concomitant treatment with the *A. maurorum* extract significantly reduced (*p* < 0.01) the expression levels of the *caspase-3* and *Bax* genes and upregulated the *Bcl-2* mRNA expression in the brain of lead + Alhagi-exposed rats compared with the lead-treated rats. The expression levels of these genes were not restored to the control levels in the lead + Alhagi-exposed group. Furthermore, the mRNA transcription of oxidative-stress-associated genes (*SOD, GPx,* and *CAT*) was significantly (*p* < 0.05) downregulated in the brain of lead-intoxicated rats relative to the control rats. These transcriptional levels were normalized by concomitant treatment with the *A. maurorum* extract in the lead + Alhagi-treated group (Figure 4).

### 3.9. Histopathological and Immunohistochemical Investigations

#### 3.9.1. Histopathological and Immunohistochemical Findings in the Liver

**Figure 5:**
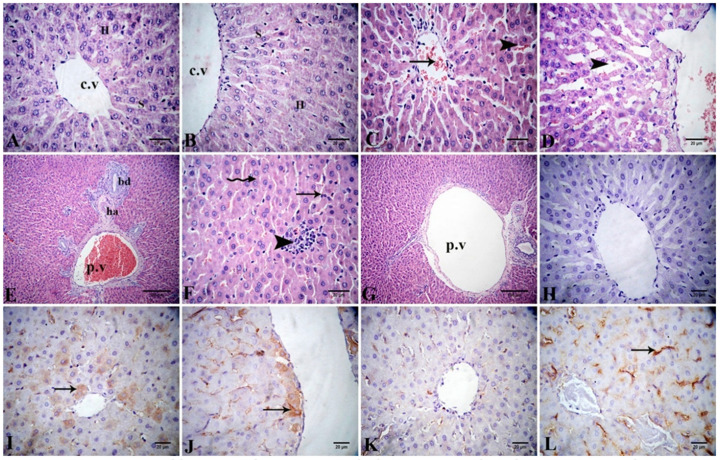
(**A**–**G**) Photomicrograph of liver tissue sections stained with H&E revealing normal histological structure of the central vein (c.v), hepatocytes (H), and sinusoids (S) in the control (**A**) and Alhagi-administered groups (**B**). The lead-intoxicated group demonstrates congestion of central vein (arrow) and in hepatic sinusoids (arrow head) (**C**), severe dilatation of sinusoids (arrow head) (**D**), congested portal vein (p.v) beside hepatic artery (ha), and bile ductule (bd) in the portal area (**E**) and lymphocytic infiltration (arrow head), disruption of hepatocytes (zigzag arrow) and apoptotic hepatocytes (arrow) (**F**). The lead + Alhagi-treated rats demonstrate mild dilation in the portal vein (p.v) (**G**). (**H**–**L**) Photomicrograph of the liver tissue sections displaying Bcl-2 immunoexpression (arrows) as the following: negative in the negative control (**H**), moderate in the control (**I**), moderate in the Alhagi-administered rats (**J**), negative to weak in the lead-intoxicated rats (**K**), and mild in the lead + Alhagi-exposed rats (**L**).

Histopathological investigation of liver sections stained with H&E from the control and Alhagi-administered rats revealed a normal histological structure of the parenchyma

(Figure 5A,B). The lead-treated group exhibited disruption of the normal arrangement of hepatocytes, with severe congestion in both the central and portal veins. Severe dilatation and congestion in the hepatic sinusoids with many apoptotic hepatocytes were observed (Figure 5C–F). In the lead + Alhagi-treated group, most of the above-mentioned lesions were reduced because the hepatic parenchyma was restored to its normal arrangement, with mild dilation of the portal vein (Figure 5G). The lesion scoring of the liver tissues in all examined groups is reported in Table 5.

The immunoexpression of Bcl-2 in the liver tissues of the various experimental groups is shown in Figure 5H–L, and its quantitative evaluation is summarized in Table 6. The liver tissues of the control and Alhagi-treated rats exhibited moderate Bcl-2 immunoexpression (26.67 ± 1.67 and 42.33 ± 5.04, respectively). In the liver of lead-treated rats, negative-to-weak immunoexpression (3.67 ± 0.67) of Bcl-2 was noticed. In contrast, co-treatment with the *A. maurorum* extract significantly (*p* < 0.001) upregulated the Bcl-2 immunoexpression to a mild level (13.00 ± 1.00) compared with the lead-exposed rats, but did not restore it to the control values.

#### 3.9.2. Histopathological and Immunohistochemical Findings in the Brain

##### Cerebral Cortex

**Figure 6:**
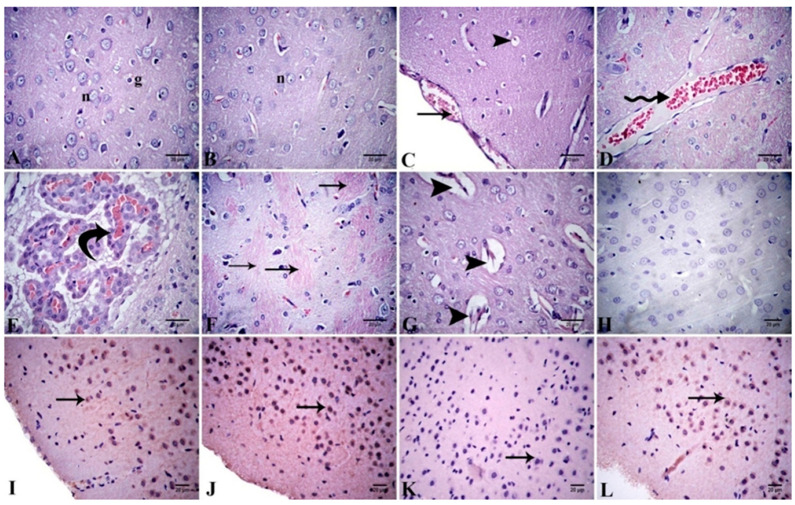
(**A**–**G**) Photomicrograph of cerebral tissue sections stained with H&E revealing normal histological structure in control (**A**) and Alhagi-administered rats (**B**), neuron (n) and glia cell (g). The lead-intoxicated rats demonstrate congested meninges (arrow) and neuropil vacuolation (arrow head) (**C**), congested blood vessel (zigzag arrow) (**D**), congested choroid plexus (curved arrow) (**E**), and demylinated nerve fibers (arrows) (**F**). The lead + Alhagi-treated rats demonstrate widening in the blood capillaries (arrow heads) (**G**). (**H**–**L**) Photomicrograph of the cerebral tissue sections exhibiting Bcl-2 immunoexpression (arrows) as the following: negative in the negative control (**H**), moderate in the control (**I**), strong in the Alhagi-administered rats (**J**), mild in the lead-intoxicated rats (**K**), and moderate in the lead + Alhagi-exposed rats (**L**).

Cerebral cortex sections stained with H&E from the control and Alhagi-treated rats exhibited a normal histological structure (Figure 6A,B). The cerebral cortices of the lead-intoxicated group showed many pyknotic and apoptotic neurons, with vacuolation of the perineural spaces. Congestions in the blood vessels and capillaries with severe congestion of the choroid plexus were also noticed. Moreover, severe demyelination of the nerve fibers was observed (Figure 6C–F). In turn, the lead + Alhagi-treated group exhibited mild vascular and degenerative changes, including a few degenerated neurons in addition to perineural and neuropil vacuolations. Widening of the blood capillaries was a commonly observed lesion (Figure 6G). The lesion scoring of the cerebral cortex in all studied groups is reported in Table 5.

The immunohistochemical detection of Bcl-2 expression in the cerebral cortex of all groups is shown in Figure 6H–L, and the quantitative determination is reported in Table 6. The cerebral cortex of the control group exhibited moderate immunoexpression (30.00 ± 2.89) of Bcl-2, whereas the Alhagi-treated rats showed strong Bcl-2 immunoexpression (60.00 ± 5.77). Lead exposure resulted in a significant (*p* < 0.001) downregulation of Bcl-2 immunoexpression to a mild level (12.33 ± 1.45) compared with the control group. Conversely, concurrent administration of the *A. maurorum* extract significantly (*p* < 0.001) upregulated the Bcl-2 immunoexpression to a moderate level (28.33 ± 1.67) compared with the lead-intoxicated rats and restored it to the normal level.

##### Cerebellar Cortex

**Figure 7:**
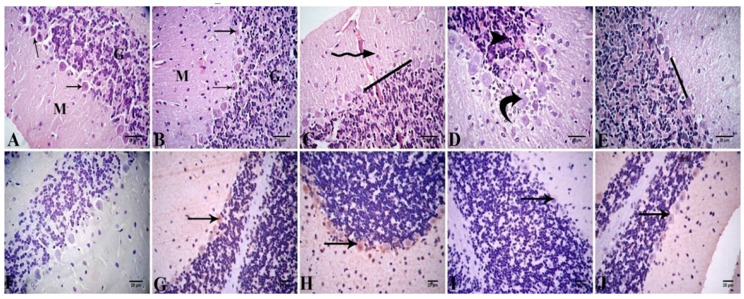
(**A**–**E**) Photomicrograph of cerebellar tissue sections stained with H&E revealing normal histological structure in control (**A**) and Alhagi-administered rats (**B**), molecular layer (M), Purkinje cell layer (arrows), and granular layer (G). The lead-treated rats show congested cerebellar cortex (zigzag arrow) and loss of Purkinje cells in some areas (____) (**C**), Purkinje cells coalesce together (curved arrow), and pyknotic nuclei of granule cells (arrow head) (**D**). The lead + Alhagi-exposed rats demonstrate loss of Purkinje cells in some areas (____) (**E**). (**F**–**J**) Photomicrograph of the cerebral tissue sections revealing Bcl-2 immunoexpression (arrows) as the following: negative in the negative control (**F**), mild in the control (**G**), moderate in the Alhagi-administered rats (**H**), negative to weak in the lead-intoxicated rats (**I**), and mild in the lead + Alhagi-exposed rats (**J**).

The light microscopical examination of H&E-stained cerebellar cortical sections from the control and Alhagi-administered rats showed a normal histological structure; the gray matter had three layers, i.e., the outer molecular layer, the middle Purkinje cell layer, and the inner granular cell layer (Figure 7A,B). In the lead-treated group, there was congestion in blood vessels, and the histopathological lesions mainly appeared on the Purkinje cell layer. Moreover, they disappeared in several areas, and their normal pyriform shape was disrupted. Many Purkinje cells aggregated with each other. The granular layer showed pyknosis of the nuclei (Figure 7C,D). The cerebella of the lead + Alhagi-treated rats revealed that the histopathological changes were attenuated, with the exception of the disappearance of Purkinje cells (Figure 7E). The lesion scoring of the cerebellar cortex in all groups is presented in Table 5.

The immunoexpression of Bcl-2 in the cerebellar cortices of all examined groups is illustrated in Figure 7F–J, and the quantitative assessment of Bcl-2 immunoexpression is displayed in Table 6. The cerebellar cortex of the control group exhibited mild immunopositive reaction (13.00 ± 1.00) for Bcl-2. The Alhagi-treated rats showed moderate Bcl-2 immunoexpression (38.33 ± 6.01) in the cerebellar cortex. Exposure to lead provoked a significant (*p* < 0.001) downregulation of Bcl-2 immunoexpression to a weak level (1.67 ± 0.33) relative to the control group. However, simultaneous treatment with the *A. maurorum* extract significantly (*p* < 0.001) normalized the immunoexpression (11.67 ± 1.67) of Bcl-2 in the lead + Alhagi-exposed rats.

##### Hippocampus

**Figure 8:**
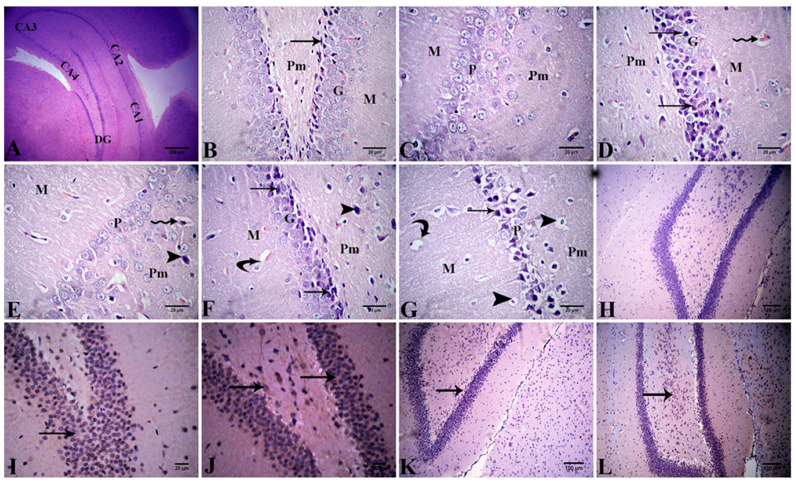
(**A**–**G**) Photomicrograph of the hippocampus tissue sections stained with H&E. CornuAmmonis (CA), dentate gyrus (DG), molecular layer (M), pyramidal layer (P), polymorphic layer (Pm), and granular layer (G) in control (**A**), and Alhagi-treated rats (**B**,**C**) showing normal structure of hippocampus, the subgranular zone of dentate gyrus (arrow) (**B**) and the normal arrangement of CornuAmmonisin (**C**). The lead treated-rats show necrotic neurons of granular layer of DG (arrows) and blood capillaries widening (zigzag arrow) (**D**), necrotic neuron of polymorphic layer of CornuAmmonis (arrow head) and blood capillaries widening (zigzag arrow) (**E**). The lead + Alhagi-treated rats show vacuolation of neuropil (curved arrows), necrotic neurons (arrows), and vacuolation of perineural spaces (**F**,**G**). (**H**–**L**) Photomicrograph of the hippocampus demonstrate Bcl-2 immunoexpression (arrows) as the following: negative in the negative control (**H**), moderate in the control (**I**), strong in the Alhagi-administered rats (**J**), negative to weak in the lead-intoxicated rats (**K**), and mild in the lead + Alhagi-exposed rats (**L**).

Histopathological investigation of hippocampal sections stained with H&E from the control and Alhagi-administered rats revealed a normal histological structure in the dentate gyrus (DG) and hippocampus proper, which consists of the CornuAmmonis (CA) CA1, CA2, CA3, and CA4 areas. Hippocampal cells were formed from three layers, including the molecular, pyramidal, and polymorphic layers. The DG was a dark C-shaped structure enclosing the CA4 area. The DG was formed from the molecular, granular, and polymorphic layers. The granular layer was composed of granule cells; its shape was rounded and it had vesicular nuclei. The subgranular zone comprised darkly pigmented cells with different shapes (Figure 8A–C). In the DG of rats in the lead-treated group, the granular layer exhibited necrotic neurons with darkly stained nuclei, and widening of blood capillaries. The pyramidal layers in the CA showed no abnormal changes; however, widening of blood capillaries in polymorphic and molecular layers was observed (Figure 8D,E). In rats treated with lead and *A. maurorum*, the hippocampus exhibited mild histopathological changes, such as few necrotic neurons, vacuolation of neuropil, vacuolation of perineural spaces, and widening of blood capillaries (Figure 8F,G). The lesion scoring of the hippocampus in all examined groups is presented in Table 5.

The hippocampal neural immunoexpression of Bcl-2 is illustrated in Figure 8H–L, and its quantitative assessment is reported in Table 6. The hippocampus of the control group showed moderate immunoexpression (30.00 ± 2.89) of Bcl-2, whereas the Alhagi-treated group exhibited strong Bcl-2 immunoexpression (60.00 ± 5.77). Exposure to lead caused a significant (*p* < 0.001) downregulation of Bcl-2 immunoexpression to a weak level (3.67 ± 0.67) compared with the control group. Conversely, concomitant administration of the *A. maurorum* extract significantly (*p* < 0.001) upregulated the immunoexpression of Bcl-2 to a mild level (13.33 ± 0.88) compared with the lead-intoxicated rats but did not restore it to the normal levels.

## 4. Discussion

Numerous natural products have been demonstrated to be highly effective in reducing the risks associated with unavoidable heavy metal exposure [5,33]. Therefore, the present investigation evaluated the efficacy of an *A. maurorum* extract in modulating the hepatotoxic and neurotoxic alterations caused by lead exposure.

The present data revealed a remarkable decrease in body weight gain in the lead-exposed group compared with the control rats. This may be attributed to a direct action of lead on the digestive tract, producing supplement malabsorption, or to a reduction in food intake, because lead affects the signals of food satiety, resulting in early meal termination [34]. These findings agreed with those of previous investigations [7,35]. However, other reports have found that lead exposure has no effect on body weight [36,37]. These discrepancies may be attributed to variations in lead exposure level and duration. Notably, concomitant treatment with the *A. maurorum* extract markedly attenuated the decrease in body weight gain.

In the present study, rats exposed to lead exhibited enhanced locomotor activity, as indicated by a significantly (*p* < 0.01) increased ambulation frequency, shorter freezing duration, and increased number of entries into the central area of the open field. Because we also noticed an increase in total locomotion, this behavior did not indicate decreased anxiety. An increase in central locomotion without a change in overall locomotion could be interpreted as an anxiolytic outcome, as noted in previous publications [7,38]. This pattern most likely indicates dopaminergic dysfunction [39]. This hypothesis was supported by the reduced dopamine level that was detected in the brain of the lead-treated group herein. This assumption is related to the fact that the dopamine neurotransmitter is implicated in many CNS activities, including cognition, mood, motor activity, learning, and attention. Therefore, any changes in its transport could have a negative impact on a wide range of neurological functions, resulting in serious behavioral abnormalities [40]. Additionally, lead had no effect on the stereotypic exploratory behavior such as grooming and rearing behaviors in the current study. Conversely, other studies have documented that lead exposure reduced both locomotor activity and stereotypic exploratory behavior [41,42]. This difference could be attributable to various exposure doses and times.

Moreover, the exposure to lead carried out in the current investigation evoked significant sensorimotor deficits, as evidenced by impaired swimming and inclined plane performance as well as reduced grip strength time. These neurobehavioral abnormalities suggest the malfunction of various anatomical regions in the skeletal muscles, CNS, or peripheral nervous system [43]. In the present work, a variety of pathological lesions were observed in the brain tissues of the lead-exposed rats, such as neuronal degeneration, neuropil vacuolation, Purkinje cell layer deterioration, and congestion in the meninges, cerebral cortices, and choroid plexuses. These degenerative changes may indicate that the abnormalities in multiple brain regions could be partly attributed to sensorimotor impairment. In this context, Abd-Elhakim et al. [44] demonstrated that pathological alterations in the brain tissue can induce neurobehavioral disorders. The current data were concordant with those of previous studies [7,45]. Indeed, lead exposure has been proven to activate protein kinase C (PKC), an enzyme that plays a crucial role in many cellular processes, including cell proliferation and central nervous system development. This is the one of the established mechanisms of lead-induced neurotoxicity [46]. 

Surprisingly, the lead-induced neurobehavioral disorders observed here were significantly (*p* < 0.05) attenuated by concomitant treatment with the *A. maurorum* extract. This effect may be attributed to restoration of brain dopamine levels to normal values and protection of brain regions against the lead-induced injury that were noted in our study. The results obtained were in line with those of previous reports showing behavioral abnormalities in response to lead exposure and their correction using antioxidant therapy [7,47,48,49].

Our data revealed a marked increment in the activities of serum liver enzymes (ALT and AST) of rats subsequent to lead treatment compared with the control rats. This increment might have been caused by the release of such enzymes from the liver into the blood circulation, suggesting hepatocellular injury [50]. The resulting hepatic injury may be linked to lipid peroxidation, which disrupts the hepatocellular membrane function and structure causing plasma membrane damage [51]. This is supported by the elevated MDA concentration (as a biomarker of lipid peroxidation) induced by ROS production as a result of lead exposure, as observed in this study [52]. The liver dysfunction noticed in our study agreed with the findings of previous studies [53,54,55]. Interestingly, simultaneous treatment with the *A. maurorum* extract mitigated the lead-induced liver damage, as evidenced by an obvious decrease in the serum ALT activity and the restoration of the serum AST activity to the normal value. The hepatoprotective efficacy of the *A. maurorum* extract could be owing to the stabilization of hepatocellular membranes via the reduction of lipid peroxidation as a consequence of its antioxidant activity, as well as suppression of free radical production by the flavonoids and phenolic content [14]. In addition, 3-methyl-2-(2-oxopropyl) furan is the primary of component of *A. maurorum* extract, according to GC-MS analysis, and has been documented for its hepatoprotective activity [56]. Our data agreed with previous reports demonstrating that an *A. maurorum* extract conferred significant protection against the liver injury caused by carbon tetrachloride [14] and norfloxacin [15].

Lead toxicity is considered to be multifactorial, although it is most commonly triggered by the oxidative stress caused by the accumulation of 5-aminolevulinic acid (ALA), which is then auto-oxidized, producing superoxide anion radicals and hydrogen peroxide radicals [57]. Lead toxicity causes mitochondrial dysfunction by increasing the intracellular calcium levels, which can contribute to the generation of free radicals [58]. Furthermore, lead toxicity causes a decrease in the activity of electron transport chain constituents, thus affecting mitochondrial energy metabolism and causing free radical production [59]. The interruption of the pro-oxidant/antioxidant equilibrium is well-known as the primary mechanism by which lead affects several tissues [60].

In the current investigation, lead exposure induced oxidative stress, as manifested in the markedly (*p* < 0.001) increased lipid peroxidation biomarker (MDA), together with the remarkable decrement in the activity of antioxidant enzymes (SOD and GPx) and GSH levels in the liver and brain of lead-intoxicated rats relative to the control rats. Moreover, a notable decline in the mRNA levels of antioxidant genes, including *GPx*, *CAT*, and *SOD*, was observed in the brain and liver of the lead-intoxicated group relative to the control group. Our results were in agreement with those of previous studies [5,7,54]. In fact, it has been hypothesized that lead toxicity activates the mitogen-activated protein kinase (MAPK) pathways, which play a pivotal role in oxidative stress [61,62]. Furthermore, Li et al. [63] demonstrated that lead mediates oxidative stress in PC12 cells via the phosphoinositide 3-kinase (PI3K)/Akt/glycogen synthase kinase 3(GSK-3β) signaling pathway. Of note, concomitant treatment with the *A. maurorum* extract significantly ameliorated the lead-mediated oxidative stress by reversing the antioxidant enzyme activities and GSH level and decreasing MDA concentration. In addition, *A. maurorum* extract co-treatment normalized the mRNA transcriptional levels of antioxidant genes in the brain and liver of lead + Alhagi-treated rats. Similarly, the *A. maurorum* extract alleviated the hepatic oxidative damage triggered by carbon tetrachloride [14] and norfloxacin [15]. This alleviative effect may be attributable to the antioxidant activity of the *A. maurorum* extract. Such activity might be ascribed to its high content with several bioactive components based on the GC-MS analysis of *A. maurorum* extract in the present investigation. For instance, beta-D-glucopyranose, 1,6-anhydro-(levoglucosan) (28.91%) had been found in *Barleria noctiflora* leaves extract and reported to exhibit antioxidant and free radical scavenging activities [64]; 4H-pyran-4-one, 2,3-dihydro-3,5-dihydroxy-6-methyl, the major bioactive component detected in the *A. maurorum* extract, had been documented to have strong antioxidant properties [65,66]; 3-methyl-2-(2-methyl-2-butenyl)-furan (rosefuran) is the main active compound identified in the *A. maurorum* extract and had been shown to have hydroperoxyl radical scavenging activity [67].

Dopamine is a neurotransmitter that plays a crucial role in different brain functions, such as cognition, control of locomotion, and emotion. In the current study, the lead-exposed group exhibited a significant decrease (*p* = 0.001) in the brain dopamine content compared with the control group. These observations agree with those of previous investigators [42,68]. However, an earlier study has reported increase in dopamine levels following lead exposure [69]. Differences in exposure levels and duration may account for this discrepancy. The reduced dopamine concentration may be attributed to the downregulation of tyrosine hydroxylase, which is a dopamine biosynthesis enzyme, resulting in a decline in dopamine production [68]. Of note, co-treatment with the *A. maurorum* extract restored the dopamine concentration to the normal level, thus reversing the neurobehavioral impairments induced by lead exposure.

The present results revealed that lead exposure triggered hepatic and neuroapoptosis, as indicated by the remarkable increase in the mRNA levels of pro-apoptotic genes (*Bax* and *caspase-3*) and the significant decrease in anti-apoptotic *Bcl-2* mRNA transcriptional levels in the liver and brain of lead-intoxicated rats. In addition, reduced *Bcl-2* immunoexpression was recorded in the liver, cerebral and cerebellar cortices, and hippocampus of rats in the lead-intoxicated group compared with the control group. These observations coincided with those of previous investigations [18,50,70]. Lead-induced apoptosis may be the result of oxidative damage, as ROS levels were dramatically elevated after lead exposure [5,71]. Apoptosis is triggered by the lead-induced hyperacetylation of histones and mitochondrial membrane disturbance, thus causing cytochrome c release [72]. Moreover, Baty et al. [73] found that the dephosphorylation of the extracellular signal-regulated kinase and the caspase cascade are the most important pathways related directly to the apoptotic signals induced by lead. Consistent with our results, previous in vivo and in vitro investigations clarified the involvement of the *caspase-3* and *Bcl-2* families in lead-mediated apoptosis [74,75]. Additionally, numerous studies have demonstrated that lead exposure triggers apoptosis through activation of tumor suppressor protein (*P53)* and *Bax* [76,77,78]. The activation of these proteins exacerbates mitochondrial dysfunction [79]. Interestingly, concomitant treatment with the *A. maurorum* extract significantly reduced apoptosis in the liver and brain tissues, as denoted by the marked downregulation of the *caspase-3* and *Bax* genes and upregulation of the *Bcl-2* gene. Furthermore, co-administration of the *A. maurorum* extract elevated the immunoexpression of Bcl-2 in the liver, cerebral and cerebellar cortices, and hippocampus of the lead +Alhagi-treated group relative to the lead-treated group. These findings indicate that the *A. maurorum* extract provoked an anti-apoptotic effect.

The results reported above were supported by the liver and brain histopathological observations performed here. Histopathological investigation of the liver and brain in the lead-treated group revealed remarkable architectural changes and extensive damage, similar to that reported in previous studies [4,50,54]. However, simultaneous treatment with the *A. maurorum* extract reversed these lesions. This indicated that the *A. maurorum* extract offers histological protection against lead-induced liver and brain injuries. These findings are supported by earlier reports that clarified that the *A. maurorum* extract exerted a protective effect on the hepatic histological injury triggered by carbon tetrachloride [14] and norfloxacin [15].

## 5. Conclusions

The present findings suggest that concomitant treatment with the *A. maurorum* extract rescued the lead-induced hepatotoxicity and neurotoxicity in rats by enhancing neurobehavioral disorders, hepatic and brain antioxidant status, and liver function; restoring the normal brain dopamine levels and the mRNA levels of antioxidant genes; and minimizing liver and brain histological changes and apoptosis. This ameliorative effect of the *A. maurorum* extract may be associated with its antioxidant and anti-apoptotic properties, as well as its capability to scavenge free radicals and abrogate lipid peroxidation. Additional studies are necessary to examine the attenuating effect of the *A. maurorum* extract regarding the liver and brain damage induced by other heavy metals.

## Figures and Tables

**Table 1:** Oligonucleotide primer sequences used for real-time PCR.

Genes	Forward Primer (5′ → 3′)	Reverse Primer (5′ → 3′)	Product Size/bp	GenBank Accession Numbers
*Bax*	CGAATTGGCGATGAACTGGA	CAAACATGTCAGCTGCCACAC	109	NM_017059.2
*Bcl-2*	GACTGAGTACCTGAACCGGCATC	CTGAGCAGCGTCTTCAGAGACA	135	NM_016993.1
*Caspase-3*	GAGACAGACAGTGGAACTGACGATG	GGCGCAAAGTGACTGGATGA	147	NM_012922.2
*CAT*	GTCCGATTCTCCACAGTCGC	CGCTGAACAAGAAAGTAACCTG	272	AH004967.1
*SOD*	ATGGGGACAATACACAAGGC	TCATCTTGTTTCTCGTGGAC	225	Z21917.1
*GPx*	CACAGTCCACCGTGTATGCC	AAGTTGGGCTCGAACCCACC	292	S50336.1
*GAPDH*	GGCACAGTCAAGGCTGAGAATG	ATGGTGGTGAAGACGCCAGTA	143	NM_017008.4

**Table 2:** Retention time and peak area sum (%) of the various compounds present in *A. maurorum* ethanolic extract analyzed by GC-MS.

Compound	Molecular Formula	RT (min)	Peak Area Sum %
Beta-D-glucopyranose, 1,6-anhydro-	C_6_H_10_O_5_	24.97	28.91
4H-pyran-4-one, 2,3-dihydro-3,5-dihydroxy-6-methyl-	C_6_H_8_O_4_	15.38	23.24
3-methyl-2-(2-oxopropyl)furan	C_8_H_10_O_2_	25.45	13.56
3-methyl-2-(2-methyl-2-butenyl)-furan	C_10_H_14_O	23.19	12.84
(3Z,6Z)-3,6-octadien-1-ol	C_8_H_14_O	28.99	7.83
Beta-l-arabinopyranoside, methyl	C_6_H_12_O_5_	25.28	7.51
Phenyllactic acid benzyl ester	C_12_H_16_O_3_	28.36	2.99
Anti-3,3-dimethyl-6-hydroxybicyclo [2.2.2] octan-2-one	C_10_H_16_O_2_	27.42	1.72
(4R,6S)-4-(t-butyldimethylsilyl)-6-fluoro-5-nonanone	C_15_H_3_1FOSi	27.23	1.40

**Table 3:** Effect of *A. maurorum* extract (300 mg/kg BW, orally) on body weight change and relative liver and brain weights in rats exposed to lead (100 mg/kg BW, orally) daily for 3 months.

Parameters			Groups		
	Control	Alhagi	Lead	Lead + Alhagi	*p*-Value
Initial body weight (g)	216.00 ± 3.06	216.67 ± 4.41	208.33 ± 4.26	222.67 ± 3.71	0.157
Final body weight (g)	307.67 ± 1.45	311.33 ± 5.21	275.67 ± 3.18 ***	308.00 ± 1.53 ^###^	0.000
Body weight change (g)	91.67 ± 1.67	94.67 ± 1.45	67.33 ± 1.45 ***	85.33 ± 2.73 ^###^	0.000
Relative liver weight (%)	2.69 ± 1.21	2.82 ± 4.41	2.50 ± 0.04	2.58 ± 0.01	0.064
Relative brain weight (%)	0.57 ± 0.01	0.56 ± 0.03	0.60 ± 0.02	0.56 ± 0.01	0.518

Data were expressed as mean ± SE, *n* = 10 for each group. *** Significantly different compared to the control group at *p* < 0.001. ^###^ Significantly different compared to the lead-exposed group at *p* < 0.001.

**Table 4:** Effect of the *A. maurorum* extract (300 mg/kg BW, orally) on liver function markers (ALT and AST), brain dopamine level, and antioxidant enzyme activities (GPx and SOD), GSH, and MDA levels in the hepatic and brain tissues of rats treated with lead (100 mg/kg BW, orally) daily for 3 months.

			Groups		
Parameters	Control	Alhagi	Lead	Lead + Alhagi	*p*-Value
ALT (U/L)	24.33 ± 2.96	30.33 ± 1.45	98.67 ± 4.49 ***	44.67 ± 2.91 ^###^	0
AST(U/L)	25.00 ± 3.51	28.67 ± 1.86	82.00 ± 3.61 ***	34.00 ± 2.08 ^###^	0
GPx (U/g tissue)					
Liver	24.51 ± 0.29	24.86 ± 0.14	16.63 ± 0.62 ***	23.89 ± 0.49 ^###^	0
Brain	21.07 ± 0.38	21.05 ± 0.53	14.00 ± 0.58 ***	19.52 ± 0.29 ^###^	0
SOD (U/g tissue)					
Liver	61.45 ± 0.53	62.51 ± 0.42	36.61 ± 0.49 ***	60.28 ± 1.23 ^###^	0
Brain	40.69 ± 0.43	40.71 ± 0.23	24.86 ± 0.81 ***	39.19 ± 0.41 ^###^	0
GSH (µmol/g tissue)					
Liver	1.33 ± 0.02	1.33 ± 0.01	0.60 ± 0.04 ***	1.28 ± 0.03 ^###^	0
Brain	3.21 ± 0.12	3.31 ± 0.12	1.63 ± 0.09 ***	2.89 ± 0.06 ^###^	0
MDA (nmol/g tissue)					
Liver	90. 33 ± 0.33	89.67 ± 1.20	183.33 ± 1.76 ***	123. 67 ± 2.03 ^###^	0
Brain	60.33 ± 0.33	59.00 ± 0.58	92.33 ± 1.45 ***	73.00 ± 1.16 ^###^	0
Dopamine (ng/mL)	26.77 ± 1.91	24.20 ± 2.98	10.25 ± 0.50 **	20.54 ± 0.87 ^##^	0.001

Data were expressed as mean ± SE, *n* = 10 for each group. **, *** Significantly different compared to the control group at *p* < 0.01 and *p* < 0.001, respectively. ^##^, ^###^ Significantly different compared to the lead-exposed group at *p* < 0.01 and *p* < 0.001, respectively.

**Table 5:** Lesion scoring in the liver and brain tissues of rats in response to lead (100 mg/kg BW, orally) and *A. maurorum* extract (300 mg/kg BW, orally) treatments daily for 3 months.

			Groups			
Organ	Lesion	Control	Alhagi	Lead	Lead + Alhagi	*p*-Value
Liver	Congestion of central vein	0 ± 0	0 ± 0	3.33 ± 0.33 ***	1.67 ± 0.33 ^###^	0
	Congestion of portal vein	0 ± 0	0 ± 0	4.00 ± 0.58 **	1.33 ± 0.88 ^##^	0.002
	Congestion of hepatic sinusoids	0 ± 0	0 ± 0	1.33 ± 0.33 *	0.33 ± 0.33 ^#^	0.012
	Dilatation of hepatic sinusoids	0.67 ± 0.33	0.33 ± 0.33	3.00 ± 0 ***	1.00 ± 0 ^###^	0
	Lymphocytic infiltration	0 ± 0	0 ± 0	2.00 ± 0.58 **	0.67 ± 0.33 ^##^	0.009
	Disruption of hepatocytes	0 ± 0	0 ± 0	1.33 ± 0.33 *	0.33 ± 0.33 ^#^	0.012
Cerebral cortex	Congestion of meninges	0 ± 0	0 ± 0	4.00 ± 0.58 ***	3.00 ± 0.58 ^###^	0
	Congestion of cerebral cortex	0 ± 0	0 ± 0	2.33 ± 0.33 ***	0.33 ± 0.33 ^###^	0
	Congestion of choroid plexus	0.33 ± 0.33	0 ± 0	4.00 ± 0.58 ***	3.00 ± 0.58 ^###^	0
	Demyelination of nerve fibers	0 ± 0	0 ± 0	4.00 ± 0 ***	2.00 ± 0.58 ^###^	0
	Vacuolation of perineural spaces	0.67 ± 0.33	0.67 ± 0.33	2.33 ± 0.33	1 ± 0.58	0.059
	Neuropil vacuolation	0 ± 0	0 ± 0	2.00 ± 0 ***	0.33 ± 0.33 ^###^	0
Cerebellar cortex	Congestion of cerebellar cortex	0 ± 0	0 ± 0	1.33 ± 0.33 *	0.33 ± 0.33 ^#^	0.012
	Disappearance of Purkinje cells	0 ± 0	0 ± 0	2.67 ± 0.33 ***	1.67 ± 0.33 ^###^	0
	Change of normal shape of Purkinje cells	0 ± 0	0 ± 0	2.00 ± 0 ***	2.67 ± 0.33 ^###^	0
	Coalescing of Purkinje cells together	0 ± 0	0 ± 0	1.67 ± 0.33 **	0.33 ± 0.33 ^##^	0.003
Hippocampus	Degenerated neurons	0 ± 0	0 ± 0	4.00 ± 0.58 ***	1.67 ± 0.33 ^###^	0
	Widening of blood capillaries	0 ± 0	0 ± 0	3.00 ± 0.58 ***	2.00 ± 0 ^###^	0
	Vacuolation of neuropil	0 ± 0	0 ± 0	3.00 ± 0 ***	1.33 ± 0.33 ^###^	0

Data were expressed as mean ± SE, *n* = 10 for each group. *, **, *** Significantly different compared to the control group at *p* < 0.05, *p* < 0.01, and *p* < 0.001, respectively. #, ##, ### Significantly different compared to the lead-exposed group at *p* < 0.05, *p* < 0.01, and *p* < 0.001, respectively.

**Table 6:** Immunohistochemical evaluation of Bcl-2 in the liver and brain tissues of rats in response to lead (100 mg/kg BW, orally) and *A. maurorum* extract (300 mg/kg BW, orally) treatments daily for 3 months.

			Groups			
Organ	Negative Control	Control	Alhagi	Lead	Lead + Alhagi	*p*-Value
Liver	0 ± 0	26.67 ± 1.67	42.33 ± 5.04	3.67 ± 0.67 ***	13.00 ± 1.00 ^###^	0
Cerebral cortex	0 ± 0	30.00 ± 2.89	60.00 ± 5.77	12.33 ± 1.45 ***	28.33 ± 1.67 ^###^	0
Cerebellar cortex	0 ± 0	13.00 ± 1.00	38.33 ± 6.01	1.67 ± 0.33 ***	11.67 ± 1.67 ^###^	0
Hippocampus	0 ± 0	30.00 ± 2.89	60.00 ± 5.77	3.67 ± 0.67 ***	13.33 ± 0.88 ^###^	0

Data were expressed as mean ± SE, *n* = 10 for each group. *** Significantly different compared to the control group at *p* < 0.001. ^###^ Significantly different compared to the lead-exposed group at *p* < 0.001.

## Data Availability

Data is contained within this article.
